# Spatiotemporal variation of mammalian protein complex stoichiometries

**DOI:** 10.1186/s13059-016-0912-5

**Published:** 2016-03-14

**Authors:** Alessandro Ori, Murat Iskar, Katarzyna Buczak, Panagiotis Kastritis, Luca Parca, Amparo Andrés-Pons, Stephan Singer, Peer Bork, Martin Beck

**Affiliations:** European Molecular Biology Laboratory, Structural and Computational Biology Unit, Heidelberg, Germany; Institute of Pathology, University Hospital Heidelberg, Heidelberg, Germany; Max-Delbrück-Centre for Molecular Medicine, Berlin, Germany; Present address: Leibniz Institute on Aging – Fritz Lipmann Institute (FLI), Jena, Germany; Present address: Division of Molecular Genetics, German Cancer Research Center (DKFZ), Heidelberg, Germany

**Keywords:** Protein complex, Stoichiometry, Proteomics, Paralog, Epigenetic, Transport, Reprogramming, Cancer

## Abstract

**Background:**

Recent large-scale studies revealed cell-type specific proteomes. However, protein complexes, the basic functional modules of a cell, have been so far mostly considered as static entities with well-defined structures. The co-expression of their members has not been systematically charted at the protein level.

**Results:**

We used measurements of protein abundance across 11 cell types and five temporal states to analyze the co-expression and the compositional variations of 182 well-characterized protein complexes. We show that although the abundance of protein complex members is generally co-regulated, a considerable fraction of all investigated protein complexes is subject to stoichiometric changes. Compositional variation is most frequently seen in complexes involved in chromatin regulation and cellular transport, and often involves paralog switching as a mechanism for the regulation of complex stoichiometry. We demonstrate that compositional signatures of variable protein complexes have discriminative power beyond individual cell states and can distinguish cancer cells from healthy ones.

**Conclusions:**

Our work demonstrates that many protein complexes contain variable members that cause distinct stoichometries and functionally fine-tune complexes spatiotemporally. Only a fraction of these compositional variations is mediated by changes in transcription and other mechanisms regulating protein abundance contribute to determine protein complex stoichiometries. Our work highlights the superior power of proteome profiles to study protein complexes and their variants across cell states.

**Electronic supplementary material:**

The online version of this article (doi:10.1186/s13059-016-0912-5) contains supplementary material, which is available to authorized users.

## Background

Recent large-scale proteomic efforts have identified proteins corresponding to more than 80 % of the human protein-coding genes, thousands of which have a restricted tissue distribution [1, 2]. Elucidating the consequences of tissue-specific protein expression is a key challenge towards understanding how proteins modulate phenotypic variation during differentiation and conduct cell-type specific functions in various (patho-)physiological settings. Protein complexes are the ultimate effectors of many biological functions, their topology has been systematically charted in both lower and higher eukaryotes [3–6], and the co-expression of their members has been investigated during the cell cycle [7, 8] and across mutant yeast strains [9] using gene expression data. However, how protein complexes are modulated by cell-type specific protein expression remains largely unknown [1]. Recently, it has been shown that protein stoichiometry can vary across cell types and temporal states, however, the limited number of investigated complexes [10–12] or investigated states [5] prompted for a more global study to generalize these findings, show robustness, and derive mechanistic insights.

Here, we globally analyze protein complex stoichiometries in mammalian cells using two publicly available large-scale proteomic datasets that resolve protein expression in space and time. The first dataset contains the proteome of 11 human cancer cell lines that represent stable differentiation states and cover the most relevant cancer types such as carcinoma, leukemia, sarcoma, and glioblastoma [13]. The second proteomic dataset covers the reprogramming of mouse embryonic fibroblasts into induced pluripotent stem cells (iPSC) and is temporally resolved over 15 days (five states in total) following the induction of the transcription factors Oct4, Klf4, Sox2, and c-Myc [12]. We found that in both settings more than 50 % of the 182 well-characterized protein complexes investigated here are subject to stoichiometric variations, and that there is a considerable overlap of complexes and complex members that are variable in space and time. Strikingly, variations occur most frequently in regulators of chromatin structure and intracellular transporters suggesting that multi-cellular organisms utilize stoichiometric fine-tuning of protein complexes not only to reshape their epigenetic landscape but also to modulate the distribution of molecules between compartments in a cell-type specific manner. We report several previously unknown paralog switches, and demonstrate that the co-regulation of paralogous proteins is a common phenomenon that requires the integration of both transcriptional and post-transcriptional mechanisms. Finally, we show that compositional signatures of protein complexes can be used to discriminate normal from cancer tissue and might hold diagnostic potential in the future.

## Results and discussion

### Coordinated expression of protein complex members across proteome profiles

**Fig. 1:**
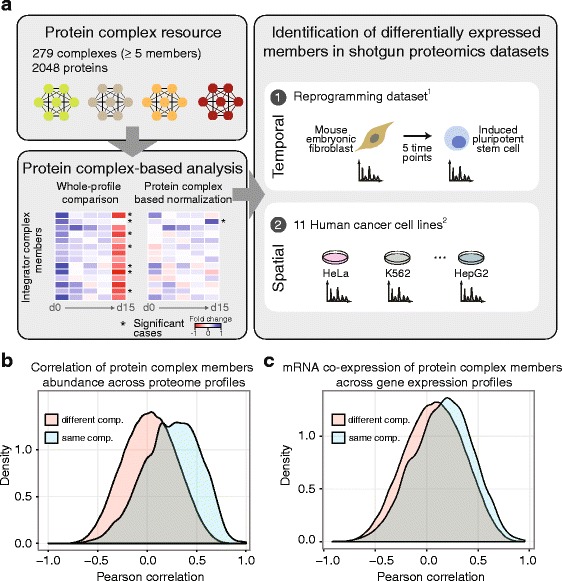
**a** Workflow used for the identification of protein complex variants from large-scale proteomics dataset. Protein complex definitions were assembled from different resources and the literature (Additional file 2, Methods). Protein complex-based normalization [10] was used to investigate compositional changes in two published datasets: a time course experiment of fibroblast reprogramming to induced pluripotent stem cells [12] and an 11 cancer cell line dataset [13]. **b** The abundance of members of protein complexes is correlated across proteome profiles. A total of 824 proteins from 123 complexes were quantified in the 11 cell lines dataset [13]. Distributions of Pearson correlation coefficients were plotted for pairwise comparisons between members of the same complex (*light blue*) and between proteins from different complexes (*orange*). **c** Similar to **b**, except co-expression between members of protein complexes was calculated using gene expression profiles derived from 10 cancer cell lines (missing GAMG cell line from Geiger et al. [13]). Co-expression analysis was limited to the set of proteins from **b** that were also quantified in the microarray experiment (HG-U133A, Additional file 3). Pearson correlation coefficients were calculated between pairs of 796 proteins from 117 complexes. Members of protein complexes show higher co-expression at the mRNA level, but to a much lesser extent than the protein co-expression

To capture as many known large complexes as possible, we generated a manually curated protein complex resource by integrating information from the following sources: (i) a compilation of literature-curated complexes; (ii) the CORUM, a comprehensive resource of manually annotated complexes [14]; and (iii) the COMPLEAT complex resource that was generated based on literature data and protein-protein interaction networks [15]. After redundancy filtering, we defined 279 largely non-overlapping protein complexes, each one composed of at least five distinct proteins (Fig. 1a, Additional file 1: Figure S1 and Additional file 2). In total, these complexes cover 2048 unique proteins, corresponding to approximately one-fifth of the proteome generally expressed by mammalian cells of a given cell type [16, 17].

Proteins belonging to the same complex tend to be generally co-regulated and, therefore, their abundances correlate across cell types. In agreement with a previous study [11], we found that protein abundances of complex members (Fig. 1b) correlate better with each other than the corresponding transcript levels (Fig. 1c and Additional file 3) indicating that other regulatory processes, such as translation [18], also contribute to the resulting protein complex stoichiometries. We next investigated whether protein complexes vary in their relative abundance across cell types, which was indeed what we observed. We analyzed the co-expression of complexes across the 11 cell lines dataset and we identified clusters of correlated protein complexes (Additional file 1: Figure S2). Strikingly, protein complexes belonging to the same cellular compartment formed highly correlated clusters (Additional file 1: Figure S2). This suggests that variations in the relative abundance of protein complexes derive, to a large extent, from morphological differences between cell types that modify the proportions between protein complexes belonging to different compartments.

### Landscape of protein complex stoichiometry variation in human cells

**Fig. 2:**
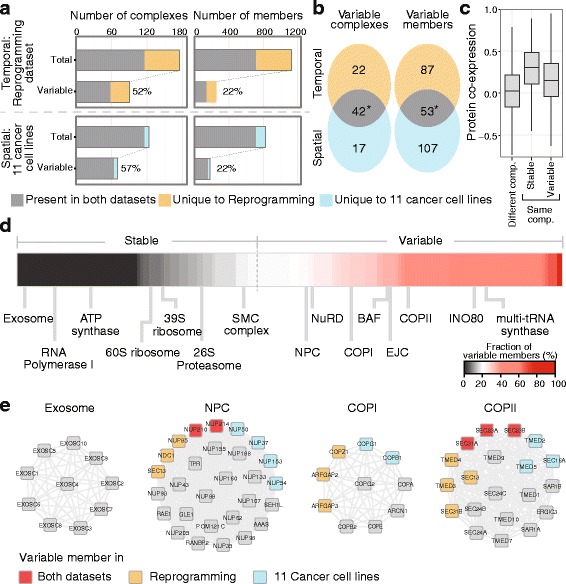
Landscape of stoichiometry variations of protein complexes. **a** About half of the investigated protein complexes undergo stoichiometric variation across cancer cell lines and during reprogramming. Proteins were defined as variable complex members if they were detected as differentially expressed (adjusted *p* value <0.05) in at least on cell line or state tested. Protein complexes were defined as variable if they had > =20 % of their members being variably expressed. Stoichiometric changes are mediated by change in abundance of a small fraction of complex members (~22 %). **b** Both variable complexes and members significantly overlap in the two datasets tested. **c** In comparison to stable members, variable members of protein complexes show significantly lower co-expression with other complex members at the protein level. **d** The fraction of variable members is higher in chromatin remodeling complexes and cellular transporters. A total of 116 protein complexes are ranked according the fraction of regulated members identified in the 11 cell lines and reprogramming dataset. **e** Selected protein complexes are shown in a network representation. Variable members are colored depending on whether they were identified as “variable complex member” in the reprogramming (*blue*), 11 cell line (*orange*), or both datasets (*red*). Stable members are shown in *gray*. Edges between nodes are based on STRING [59]

In order to study in greater detail the composition of protein complexes and to identify complex members that deviate from the general pattern of co-regulation, differences in overall complex abundance across cell types and states need to be normalized. For this purpose, we improved a previous computational method that normalizes the median complex abundance across samples prior to differential expression analysis [10] (Methods) and we applied it to globally investigate compositional changes of protein complexes across the 11 cancer cell lines and the reprogramming dataset. Of the 279 curated complexes, 182 were detected in either the 11 cell lines or the reprogramming dataset and 116 of them in both (Fig. 2a). We found that in both datasets, 22 % of the protein complex members were differentially expressed (variable complex members) in at least one of the conditions tested (adjusted *p* value <0.05) while the majority (78 %) were core complex members that remained invariant in their relative abundances (Fig. 2a and Additional file 4). As expected, stable complex members display higher correlation across proteome profiles than variable one (Wilcoxon rank sum test: *p* value <2.2E-16, Fig. 2c). To exclude potential technical biases in our analysis, we generated a decoy set of protein complex definitions by randomly assigning proteins to complexes while preserving the pool of members and the size of protein complexes. We found that while the number of identified variable complex members saturates with real complexes, it linearly increases with the number of conditions analyzed in case of the decoy set (Additional file 1: Figure S3). We thus conclude that our method robustly identifies properties of the protein complexes under investigation.

More than half of the quantified complexes had at least 20 % of their members differentially expressed in one of the investigated cell type or state, whereby half of these were common to both datasets at the complex level (Fisher’s exact test: *p* value 3.8E-4, odds ratio 3.9) as well as at the complex member level (21 % overlap, Fisher’s exact test: *p* value 1.7E-06, odds ratio 2.7, Fig. 2b). This indicates that the same complexes and complex members have a tendency to be regulated both in space and time, presumably because of their functional role in regulating the cell state and structural requirements for their assembly (for exceptions such as complexes that change stoichiometry only during reprogramming see Additional file 1: Figure S4).

### Transporters and chromatin regulating complexes are highly variable while mitochondrial complexes are stable

In order to identify functional modules that are affected by compositional changes of protein complexes, we analyzed the ratio of core to variable members across functional categories. We considered complexes as either stable or variable based on the fraction of members that was observed as differentially expressed, and we found that the majority of the analyzed complexes (102 out of 182, 56 %) were identified as variable (Fig. 2d and Additional file 4). Since we used a conservative criterion to define complexes as variable (see Methods for details) and only a limited set of cell types and states was analyzed, we expect this fraction to be possibly even larger if additional cell types and states would be compared. Out of the 182 complexes, only 80 complexes (44 %) were identified as stable (Fig. 2d and Additional file 4). Not unexpectedly, the stable complexes are enriched for Gene Ontology terms related to housekeeping biological processes such as transcription, RNA processing and translation, and energy production (Additional file 5), including e.g., RNA polymerase I and the exosome (Fig. 2e). Notably, for the cytosolic ribosome we identified few variable complex members (only 8 out of 82 of the quantified ribosomal proteins), at least one of these (RPL38) has been previously shown to have tissue-specific expression and to be able to affect the translation of specific transcript in a tissue-specific manner in mice [19], and another (RPL22L) has been shown to be differentially expressed across tissues in *Drosophila Melanogaster* [20]. Mitochondrial protein complex stoichiometries appear highly static: several components of the respiratory chain including the cytochrome bc1 complex (complex III), the cytochrome c oxidase (complex IV) and the F_0_F_1_ ATP synthase (complex V) showed stable expression of their complex members across all the 16 conditions tested (Additional file 4).

In contrast, the 102 variable complexes (Fig. 2d and Additional file 4) are enriched for regulators of chromatin structure and epigenetic modifications including, for example, the well characterized BAF, NuRD, and INO80 complexes (Additional file 5). Strikingly, the functional categories most enriched for variable complexes were related to intracellular transport of both protein and RNA (Additional file 5), including the previously described nuclear pore and TRanscription-Export (TREX) complexes [10, 12, 21]. In addition to nuclear-cytoplasmic, also vesicular transport complexes appear to be largely variable, exemplified by compositional rearrangements in COPI and COPII, the adaptor-related protein complex 3, retromer, exocyst, and SNARE complex (Fig. 2e). We therefore conclude that cell-type specific alterations of epigenetic regulators and transport systems are more frequent as compared to other functional modules in the cell.

### Both transcriptional and post-transcriptional mechanisms regulate stoichiometric variation

**Fig. 3:**
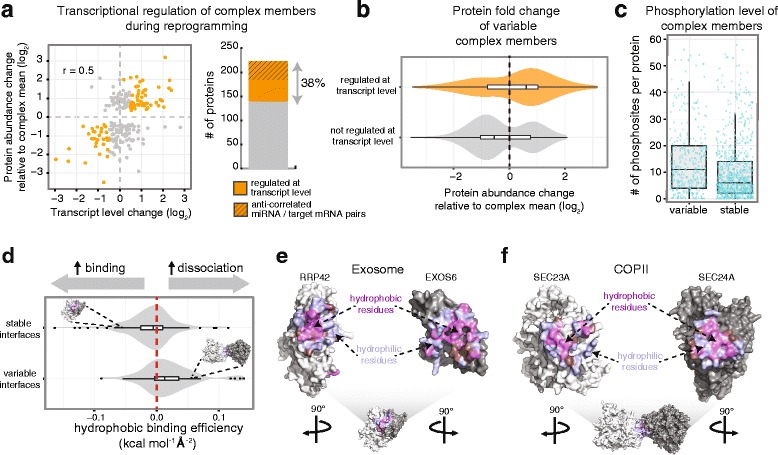
Regulation of variable complex members. **a** Co-regulation of protein abundance change and transcript expression was investigated for the reprogramming dataset using published data derived from the same time course experiment [12, 22]. Transcriptional regulation was inferred when both protein and mRNA were significantly regulated with a consistent fold change at the same time point of reprogramming. Thirty-eight percent of the investigated protein changes (223 in total) co-occur with changes in mRNA level (*orange dots*), indicating a transcriptional regulation of complex member abundance. For about half of these cases, the change in transcript level is anti-correlated with the expression of at least one miRNA targeting the same mRNA [22]. The regulation of these complex members might therefore be mediated by a miRNA-based mechanism. **b** Changes of complex members that are not transcriptionally regulated often occur via a decrease in protein abundance. *Violin plots* show the distribution of protein fold changes detected for variable complex members. Proteins are grouped according to whether transcriptional regulation could be inferred from mRNA expression data, as shown in **a**. **c** Variable complex members tend to be more phosphorylated than stable one. Phosphosites were derived from PTMcode [60]. **d** *Violin plots* for core and variable interfaces show the distribution of desolvation energies per square angstrom of apolar buried surface; stable interfaces have more favorable hydrophobic interactions (Wilcoxon rank sum test, *p* value = 8.8E-6). See Methods for details. **e** Core interface of two exosome components have hydrophobic residues that are evenly distributed in the interface core and surrounded by hydrophilic residues leading to favorable hydrophobic interaction. **f** Variable interface of two COPII components: here, hydrophobic and hydrophilic residues are mixed in the interface, leading to unfavorable hydrophobic interactions. Interfaces shown in **e** and **f** include residues within 6 Å of inter-atomic distance

We next asked whether the abundance of variable members is transcriptionally or post-transcriptionally regulated. We tackled this question using exclusively the reprogramming dataset because mRNA and miRNA expression data were available [22]. We observed an overall positive correlation between changes in protein abundance and transcript level (Pearson r = 0.5, Fig. 3a) indicating a significant degree of transcriptional regulation of compositional changes. We found that in 38 % of all variant cases (84 out of 223 members analyzed) the protein and transcript abundance changed consistently, that is into the same direction at the same time point (Fig. 3a and Additional file 6). We define such changes of stoichiometry as transcriptionally regulated. For 38 of these cases (17 % in total), miRNA expression patterns might explain the abundance variability of complex members (Fig. 3a and Additional file 6). However, a direct causality needs to be further explored. The transcriptionally regulated changes of stoichiometry most often caused an increased abundance of complex members (Fig. 3b). Vice versa, non-transcriptionally regulated compositional variations (139 cases, 62 %) most often resulted in decreased protein abundance (Fig. 3b), suggesting the involvement of other processes affecting protein turnover. Additionally, we found that variable members of protein complexes tend to be more phosphorylated as compared to stable ones (Wilcoxon rank sum test: *p* value 2.7E-9, Fig. 3c), suggesting that also post-translational mechanisms might regulate their protein levels. Taken together these findings indicate that the regulation of protein complex stoichiometries occur at multiple levels including transcription, translation, and protein turnover.

Mechanisms such as protein stabilization upon binding or competition for interfaces were previously shown to influence protein complex stoichiometry [23, 24]. We therefore asked whether protein-protein interfaces formed by variable complex members have distinct structural properties. For this purpose, we retrieved nearly 200 biological interfaces from the Protein Data Bank that covered 28 of our complexes. To systematically assess the mode of binding within these interfaces we applied established energy and accessibility calculation protocols [25] (Methods). We found that variable interfaces are significantly less hydrophobic. The binding energy per apolar surface area (kcal mol^−1^ Å^−2^) is smaller in core interfaces as compared to the interfaces formed by regulated complex members (Fig. 3d, f). None of the other investigated interface properties, namely van der Waals interaction energy, electrostatic energy, and buried surface area size, was found to significantly discriminate the two modes of binding. We thus conclude that interfaces between stable members have a tendency to be stabilized in a similar manner to the hydrophobic core of protein domain folds, while variable interfaces might be more easily accessible to regulation, e.g. by protein degradation.

### Paralog switching is a widespread mechanism that modulates protein complex composition

**Fig. 4:**
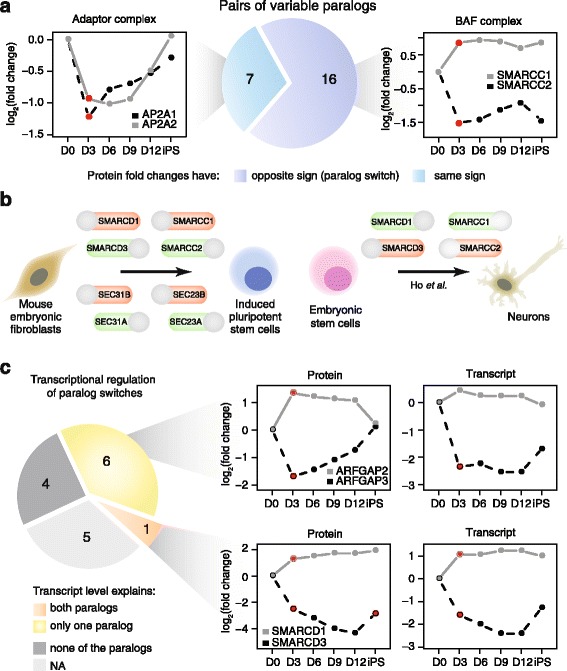
Paralog switches often mediate protein complex composition during reprogramming. **a** Twenty-three paralog pairs were found to be co-regulated at least at one time point. The majority of them had fold changes of opposite sign indicating paralog switches. Protein profiles across the five time points of the reprogramming are shown for two representative examples. *Dots* indicate the average value of independent reprogramming experiments performed in two replicates for protein profiles [12] and one to three replicates (per time point) for gene expression profiles [22]. *Red dots* indicate significant cases (adjusted *p* value <0.05, see Methods for details). *D* indicates the number of days after the induction of the reprogramming factors (D0). **b** The same paralog switches in the chromatin remodeling complex BAF that we identified during reprogramming were also found to be affected during stem cell differentiation by Ho et al. [27]. We identified 14 additional paralog switches affecting among others the COPII complex. *Red* and *green* colors indicate up- and downregulated complex members, respectively. **c** Protein abundance and mRNA expression changes were compared for the co-regulated paralogs. Transcriptional regulation was inferred when both protein and mRNA were significantly regulated with a consistent fold change at the same time point (see Methods for details). Protein and mRNA profiles are shown for two representative cases

With a large set of variable complexes and respective protein members in hand, we sought to identify common patterns that facilitate stoichiometric variations of complexes and might have been developed during the evolution of multicellular organisms. We found that complex members that have been duplicated during evolution (paralogs) are significantly enriched among the variable complex members (Fisher’s exact test: *p* values of 9.0E-6 and 6.5E-3 for reprogramming and 11 cell lines datasets, respectively). During reprogramming, we identified 23 paralogs pairs that were co-regulated at the same time point, and 16 of these (70 %) showed similar abundance differences into opposite directions (Fig. 4a and Additional file 7). Those cases likely comprise paralog switches involving mutually exclusive complex members that are antagonistically incorporated into distinct variants of the same complex [26].

Similar to other compositional changes, paralog switches affect predominantly chromatin regulators and protein complexes involved in transport systems. We identified two paralog switches in the chromatin remodeling complex BAF involving the paralogs SMARCC1/SMARCC2 and SMARCA1/SMARCA2 that co-occur within the first 3 days of reprogramming (Fig. 4b). Additionally, several switches that are induced concomitantly at the beginning of reprogramming occur in complexes involved in vesicular protein transport, including the COPI, COPII, and SNARE complexes (Additional file 7). In particular, COPII undergoes two co-occurring switches between the paralog pairs SEC23A/SEC23B and SEC31A/SEC31B (Fig. 4b). Are these events required for reprogramming to occur or are they just a consequence of the phenotypic changes induced by reprogramming itself? Interestingly, paralog switches affecting the same members of the BAF complex were previously reported to be required for maintaining pluripotency in embryonic stem cells (esBAF) [27] (Fig. 4b) and the depletion of SMARCC2 was shown to promote reprogramming [28], highlighting the central role of these proteins in promoting and maintaining a “stem-like” state. Similarly, SEC31B, but not its paralog SEC31A, was identified as a barrier to reprogramming in a large-scale RNAi screen [29]. The replacement of SEC31B with SEC31A that we observed at the beginning of reprogramming might thus represent a critical step toward the generation of iPSC. In conclusion, our data suggest that variations in the relative abundance of the two paralogs might alter the equilibrium between variants of the same complex, ultimately modulating its function, and that these phenomena are required for the efficient reprogramming of fibroblast to iPSC.

Next, we asked whether paralog switches are transcriptionally driven. For the majority of the paralog switches for which we had both proteomics and transcriptomics data (6 out of 11), we observed that changes in transcript and protein abundance correlate only for one of the two paralogs (Fig. 4c and Additional file 7). Only one pair (SMARCD1 to SMARCD3 paralog switch in BAF complex) displayed a consistent change of transcript and protein abundance for both paralogs (Fig. 4c and Additional file 7). We thus hypothesize that positively regulated paralogs might be stabilized by integration into the relevant protein complex when the paralogous partner is downregulated.

**Fig. 5:**
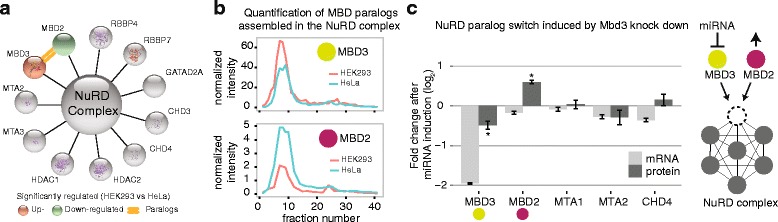
The NuRD complex undergoes a paralog switch of its members MBD2 and MBD3 across HeLa and HEK293 cells. **a** We directly compared the abundance of NuRD components between HeLa and HEK293 cells and identified a paralog switch involving MBD2 and MBD3. This suggests a different equilibrium between the MBD2- and the MBD3-containing NuRD variants in these two cell lines. Variable components are depicted as *colored circles* while core components are represented as *gray circles*. A *double orange line* connects the co-regulated paralog pair. **b** Nuclear extracts from HEK293 and HeLa cells were separated by size-exclusion chromatography and NuRD complex members were quantified by targeted proteomics (Additional file 8, Methods). As predicted by the computational analysis, MBD3 is more abundant in HEK293 cells. Since MBD2 and MBD3 are mutually exclusive in NuRD, this suggests a different equilibrium between the two variants of the complex in these cell lines. **c** *Barplot* showing the quantification of NuRD components upon induction of the miRNA targeting MBD3. mRNA levels and protein abundances were compared by qPCR and targeted proteomics upon induction of the miRNA targeting MBD3 or a scrambled miRNA control (n = 3 for each sample group). Significant protein changes (adjusted *p* value <0.01) are highlighted with an *asterisk*. The cartoon summarizes the concept that the abundances of the mutually exclusive NuRD components MBD2 and MBD3 are co-regulated: the depletion of MBD3 causes an increased abundance of its paralog MBD2 at the protein level. *Gray circles* represent core NuRD components

In order to experimentally validate this concept, we focused on the NuRD chromatin-remodeling complex as a case in point. Our computational analysis suggested that only a minority of 75 out of 1177 complex members investigated are differentially expressed between HeLa and HEK293 cells. These results are consistent with a previous biochemical fractionation study that identified only minor compositional variances across those two cell types [5]. Among the variable complexes, we identified a switch between the NuRD members MBD2 and MBD3 (Fig. 5a) and confirmed the higher abundance of MBD3-contaning NuRD complexes in HEK293 cells using biochemical fractionation and targeted proteomics (Fig. 5b and Additional file 1: Figure S5, Methods). Since this result obtained on isolated complexes exactly recapitulated the data derived from total cell extracts, it demonstrates that the majority of the expressed proteins are indeed complex associated. We next artificially reverted the MBD2/MBD3 paralog switch through inducible expression of a synthetic miRNA that reduced the abundance of MBD3 on both transcript and protein level (Fig. 5c and Methods). As a consequence, MBD2 abundance was increased on the protein but not the transcript level, while the expression of the other NuRD members remained stable (Fig. 5c). Taken together these data show that the results of our large-scale analysis are consistent with experimental validation on isolated protein complexes and confirm that the abundance of paralog proteins belonging to the same complex is often controlled by a combination of different regulatory processes.

### Protein complex composition is a signature of cell identity

**Fig. 6:**
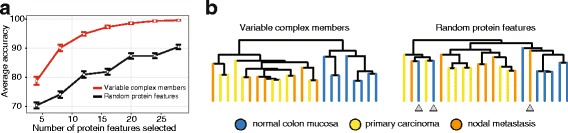
Compositional signatures discriminate between normal and cancer tissues. We used the 53 complex members that were identified as variable in both the 11 cell lines or reprogramming dataset to query an independent proteomic dataset obtained from human colon tissue samples [30]. **a** A nearest centroid method [58] was used to classify 14 cancer and seven normal tissues. Variable complex members have a better discriminative power than random protein features. Average accuracy was measured for 100 feature sets randomly sampled from variable complex members (n = 53) versus all quantified proteins (n = 6148). The size of the feature set was increased from 4 to 28 in steps of 4. The average accuracy for the variable complex members (*red*) were significantly higher in comparison to randomized features (*black*) (e.g. for n = 20, Wilcoxon rank sum test, *p* value = 9.9E-24). *Error bars* represent standard error. **b** For variable complex members and random proteins, two set of features (n = 20) were selected as representing the average accuracy. Cancer and normal proteomic profiles with the representative features were grouped by hierarchical clustering (average-linkage, using Euclidean distance) and presented as dendrograms for variable complex members and random protein features. *Gray arrowheads* indicate wrongly classified samples

The analysis of 11 distinct cell lines revealed that stoichiometric variations of protein complexes occurred consistently across human cancer cell types. Thus, we tested whether the abundance of variable complex members can be used to distinguish normal from cancer tissues. We used the complex members that were identified as variable in both the 11 cell lines and reprogramming dataset to query an independent dataset of human tumorous and non-tumorous colon tissue samples [30] (Methods). Indeed, protein features derived from the intensity of variable complex members robustly discriminated between samples of normal colon mucosa and samples of colon adenocarcinomas (primary tumors or metastases) (Fig. 6a). This very small pool of variable complex members had a comparable discriminative power as the whole proteome profiling dataset containing 7576 proteins [30]. In contrast, the same number of randomly selected protein features did not have the same discriminative power (Fig. 6b) in colon cancer. Exemplified by this highly prevalent tumor entity, our results highlight that stoichiometric variations of protein complexes occur in the course of (early) tumorigenesis and are maintained upon metastatic spreading.

## Conclusions

Here, we have quantified the co-expression of mammalian protein complex members across various cell types and states in two large-scale quantitative proteomics datasets. We selected these two datasets because they both provided high proteome coverage (>6000 protein groups) and they included multiple biological replicates for the same cell type/state. Based on the high quality of the analyzed data and the robust benchmarking of our method, we suggest that spatiotemporal modulation of molecular machines through stoichiometric variations is the norm and not the exception across mammalian cell types and states. We demonstrate that the majority of the detected stoichiometric variations are not reflected by changes in transcript levels, which might explain why they have escaped previous high-throughput gene expression analyses.

Our analysis reveals a different degree of compositional variations that segregate with the complex function. At one end, mitochondrial complexes involved in energy production were found to be highly static. Although many mitochondrial proteins are encoded in the nucleus, their independent inheritance, long evolutionary history, and their essential functional contribution to cellular homeostasis, might have prevented the evolution of stoichiometric variations in this organelle. At the other end, protein complexes involved in chromatin remodeling and cellular transport were found to be among the most variable. Why chromatin regulators and transporters appear to be among the most variable complexes? A simple explanation for this might be that both sets of complexes control the expression or the localization of a large number of molecules. Epigenetic changes are known to occur across cell types and mediate the activation of specific transcriptional programs (involving hundreds of genes) that instruct cell fate [31]. Similarly, extensive changes in the composition of the cell surface proteome have been described during differentiation [32] and reprogramming [12]. Alterations of the transport machineries could favor these remodeling events by changing the specificity of the transport systems [33]. It is tempting to speculate that cells utilize specific compositional changes of chromatin regulators and transporters to induce broad downstream effects on the proteome that are required to mediate phenotypic changes.

We further demonstrate that a reoccurring pattern is the utilization of paralogs that are mutually exclusive in complexes. They frequently have different expression behavior across cell types and states and thus replace each other in complexes. The usage of duplicated complex members as a mean to fine-tune the function of molecular machines has a long evolutionary history [34]. Already in yeast, non-redundant function and asymmetric expression profiles have been described for multiple duplicated yeast ribosomal subunits [35, 36]. The same concept might apply to other functional modules of the cell since an antagonistic expression of evolutionary related proteins was observed also for other cases during reprogramming [12]. The abundance of paralogous complex members appears to be linked, suggesting that it is tightly controlled. Since this is often not reflected at the transcriptional level, one might speculate about the existence of feedback mechanisms acting at the protein level such as protein stabilization upon complex binding.

Finally, the set of detected complex stoichiometries appears to imply higher order functionality as it is sufficient to discriminate cancer cells from benign ones. Signatures of protein complex stoichiometries may therefore hold a great potential as diagnostic markers in the future, e.g. to distinguish cancer (sub-) types or to define the tissue of origin in cancers of unknown primary. More cell types and states need to be characterized to decipher the mammalian complex landscape and might reveal many other higher order characteristics that can be predicted from a given set of complex stoichiometries.

## Methods

### Integration of a comprehensive resource of protein complexes

To systematically examine the co-expression of protein complex members, we first assembled an extensive dataset of mammalian protein complexes by integrating various large-scale resources. In order to gain sufficient statistical power in the normalization procedure, our analysis was carried out on protein complexes having at least five members. Initially, a manually curated set of 57 large protein complexes was obtained from our previous publication [10]. These complexes were further revised and with the inclusion of additional complexes, the in-house dataset was increased to 64 manually curated complexes. Next, we acquired 365 manually annotated protein complexes from the core non-redundant set of the CORUM database (downloaded from http://mips.helmholtz-muenchen.de/genre/proj/corum/) [14]. Last, the COMPLEAT protein complex resource (http://www.flyrnai.org/compleat/) was included in this study [15]. The latter resource contains 9703 human protein complexes that were either derived from literature or predicted from protein-protein interaction networks. Here, we only retained 332 reliable large complexes (> = 5 members) based on literature evidences derived from “PINdb” [37] and “CYC2008” (except “predicted”) [38] while discarding the rest. To eliminate redundant complexes in the combined dataset, we employed an iterative procedure as similarly described [15] (Additional file 1: Figures S1A and B). At the first stage, the complexes were ranked according to their source in the following order: manually curated complexes from Ori et al., COMPLEAT, and CORUM. Then, the complexes were sorted within each group according to the number of their members from largest to smallest. In a sequential order starting from first to last, we selected the highest-ranked complex as the representative and removed all complexes that shared 50 % and more of their members with this representative complex. This procedure was iteratively run till the end of the list. In total, 279 non-redundant protein complexes, having 2010 distinct members, were obtained for further analysis (Additional file 1: Figures S1C, S1D and Additional file 2). The filtering procedure used here did not take into account the proteomic data analyzed. We decided to define protein complexes a priori in order to be able to directly compare the co-expression of protein complex members across the two datasets analyzed (see below).

### Large-scale proteomic dataset

Two large-scale shotgun proteomic datasets were used in this study. The first dataset was taken from Hansson et al., a time series proteomic experiment (referred to as “reprogramming” dataset) that profiles the proteomic changes occurring through the reprogramming of mouse embryonic fibroblasts to iPSCs [12]. The reprogramming dataset contains expression changes for 5451 proteins measured between six consecutive time points (day 0 – fibroblast – to day 15 – iPSCs, profiled at 3-day intervals) in two replicates. For our analysis, we used the expression changes that were reported as protein ratios between two consecutive time points in the original publication [12]. The second dataset consists of the proteomic profiles of 11 human cell lines generated by Geiger et al. [13] (referred to as “11 cell lines” dataset). From this dataset, we retained only the 3250 proteins that were quantified in at least two out of three replicates for all the 11 cell lines and the rest was discarded. For all the cell lines we retained all the three replicates with the exception of A549 and K562, for which single replicates were identified as outlier by hierarchical clustering and excluded. For our analysis, we used the estimated protein abundances that were reported as intensity Based Absolute Quantification (iBAQ) scores in the original publication [13]. In the next step, we checked which protein complexes were represented in either of the proteomic datasets by having at least five quantified members. For reprogramming datasets, protein complexes were mapped to mouse orthologs using the Ensembl orthology data [39, 40] using the R biomart package [41]. In the end, the analysis was performed on 175 complexes, comprising 1129 proteins from reprogramming dataset, and on 123 complexes, comprising 824 proteins from 11 cell lines dataset (Fig. 2a).

### Gene expression dataset

The cell line annotation from Gene Expression Atlas [42] was used to select three replicate microarrays (except Jurkat with two replicates) for 10 cell lines (as used in Geiger et al. [13] but missing GAMG cell line). In addition, manual annotation was performed to include data for the cell lines with no available microarray experiment [43]. Randomly selected microarray experiments (listed in Additional file 3) were pre-processed using RMA normalization [44].

### Identification of differentially expressed protein complex members

To investigate compositional rearrangements of protein complexes rather than changes in overall complex abundance, we adapted a two-step normalization method that we described previously [10]. For both the reprogramming and 11 cell lines datasets, the same analysis was separately carried out as follows: individual proteome-wide profiles were median-centered, followed by outlier removal as detailed above. Subsequently, the proteomic profiles were restricted to the proteins annotated to be part of protein complexes. In agreement with our previous work [10], we found that complex members were globally co-expressed across samples (Fig. 1b). Therefore, in case of a general change in complex abundance, the comparison between samples would reveal all members to be differentially expressed (e.g. as described in [12]). In order to reveal compositional changes rather than overall complex variations, we performed an additional complex-wise normalization procedure [10]. First, relative abundances of proteins were calculated with respect to their trimmed mean across all conditions. As a next step, the abundance value of each protein was corrected by subtracting the mean relative abundance of the rest of the complex members. In case of proteins involved in multiple complexes, the average value from all the corresponding complexes was taken into consideration. After complex-wise normalization, each condition (reprogramming time point or cancer cell line) was compared with the rest of experimental conditions to identify differentially expressed complex members by LIMMA (LInear Models for Microarray data Analysis) [45]. *p* values were adjusted for each experimental condition using false discovery rate (FDR) as described by Benjamini and Hochberg [46] and members were considered as differentially expressed if the adjusted *p* value was less than 0.05 in at least one of the conditions tested. Protein complexes were considered as “variable” or “stable” depending on the fraction of members that was observed as differentially expressed relatively to the other members. To avoid an inflation of variable complexes by experimental noise, we employed a stringent threshold that requires a complex to contain at least 20 % of differentially expressed members in order to be considered as “variable complex.” Fisher’s exact test was used to assess the significance of the overlap of both variable complex members and complexes between the datasets of reprogramming and 11 cell lines (Fig. 2b).

### Analysis of protein-protein interfaces

#### Retrieval of protein-protein interfaces from the Protein Data Bank

The Protein Data Bank (PDB) structures of 281 protein-protein interfaces involving members of protein complexes included in our resource were derived using the UniProt annotation of the complexes members. All the interfaces structures were checked for quality controls: (i) the interacting proteins must be part of the biological assembly associated to the protein complex in the PDB structure; (ii) the interaction surface must be larger than 400 Å (buried surface area), this value was selected since it represents a valid lower-threshold for the association of biologically meaningful protein assemblies [47] (see below for the method used to calculate buried surface area); (iii) the proteins in the structures must be long, at least 20 amino acids. Since the protein-protein interaction can be represented by multiple PDB structures, the representative PDB entry was chosen as the one with the highest buried surface area. The final dataset composed of 184 protein-protein interfaces was analyzed as described below.

#### Buried surface area calculations

NACCESS 2.1.1 was used for accessibility calculations (http://www.bioinf.manchester.ac.uk/naccess/). In detail, we calculated the atomic accessible surface defined by rolling a probe of 1.4 Å size around the van der Waals surface of the binary protein complex [48]. We also applied the same to the separate components and then calculated differences in accessibility from the unbound to the bound state. The surface was defined by default van der Waals radii [49]. We calculated the apolar and polar buried surface areas, defined by the sum of surface accessibilities from N, O and C, S atoms, respectively. We then defined core and variable interfaces as follows:

- Variable are interfaces in which at least one partner has been shown to be differentially expressed in at least one of the condition tested;
- Core are interfaces in which both partners have been shown to be stably expressed.

Given the aforementioned conditions, we concluded that 184 complexes are suitable for subsequent energy calculations and proper analysis of the core and variable classes.

#### Energy calculations

In order to ensure that all potentially missing side-chains were properly built and the interface optimized, the HADDOCK webserver refinement protocol was used [50], first described by Kastritis and Bonvin [51]. We used the OPLS force field [52]. Non-bonded interactions were calculated using a cutoff of 8.5 Å. A shift function was applied for calculating Electrostatic energy (E_elec_), while a switching function (between 6.5 and 8.5 Å) was applied for the calculation of van der Waals interaction energy (E_vdw_). Implementation of empirical atomic solvation parameters were used for Desolvation energy calculation (E_desolv_) using parameters from Fernandez-Recio et al. [53]). This procedure generated 50 refined protein-protein interfaces per complex, starting from different random velocities. As is default in the HADDOCK protocol, the average score of the top four models was evaluated. All calculations were performed with HADDOCK version 2.1/CNS version 1.2 [54] through the refinement interface of the HADDOCK web server (http://haddock.science.uu.nl/). Details on the protocol have been previously described and can be found in [51] and [55].

### Regulation of protein complex stoichiometry

For the reprogramming dataset, mRNA and miRNA expression profiles performed on the same time course experiment as the proteomics data were retrieved from Polo et al. [22]. These datasets were downloaded from the GEO database with the accession number GSE42478. Relevant gene expression profiles were normalized with RMA procedure and LIMMA analysis was used for the comparison of consecutive time points in order to identify differentially expressed probe sets (FDR adjusted *p* value <0.05 and absolute log_2_ fold change >0.5). The comparison between “day12” (GEO accession: GSM1038611) and “day9” (GEO accession: GSM1038607) could not be undertaken because both these time points had only single replicates (in order to generate the graphs displayed in Fig. 4c we therefore assigned a fold change with value 0 to this time point). In total, 9183 out of 22,716 probe sets were found to be significant in at least one of the time points. Only the probe set with highest variance was selected to avoid bias towards genes represented by multiple probe sets. Next, we compared the protein abundance profiles to changes in transcript expression across the time course experiment for differentially expressed complex members (Additional file 6). For 71 out of 223 analyzed cases for which we had complete data, we found significant and consistent (same sign) changes at both the protein and mRNA level that were co-occurring at the same time point during reprogramming (indicated as “TRUE” in Additional file 6). For additional 13 cases, the protein change was accompanied by a consistent trend at the mRNA level (absolute log_2_ fold change >0.5, FDR adjusted *p* value >0.05, indicated as “TREND” in Additional file 6). We interpreted both such cases as evidence that the abundance of complex member is regulated at the transcriptional level (Fig. 3a and b). Additionally, we retrieved the predicted mRNA targets of significantly regulated miRNAs (LIMMA, FDR adjusted *p* value <0.01) from targetscan database [56], as similarly done in Polo et al. [22]. Finally, differentially co-expressed mRNA/proteins were linked with inversely expressed miRNAs and these cases were indicated as potentially mediated by miRNAs (Fig. 3b).

### Analysis of NuRD composition in HeLa and HEK293 nuclear extracts

Nuclei were isolated from HeLa and HEK293 cells as described in [57]. All the following steps were performed at 4 °C, unless otherwise stated. Nuclei were resuspended at concentration between 1.5e8/mL and 3.0e8/mL in digestion buffer A (0.1 mM MgCl_2_, 1 mM DTT, 10 μg/mL aprotinin, 5 μg/mL leupeptin) supplemented with DNaseI (Roche, cat.n: 104145) and RNAseA (Sigma, R4642), and immediately diluted with 4 volumes of digestion buffer B (5 % (v/v) glycerol, 20 mM Tris–HCl pH 8.5, 0.1 mM MgCl_2_, 1 mM DTT, 10 μg/mL aprotinin, 5 μg/mL leupeptin). DNA and RNA digestion was allowed to proceed for 15 min at room temperature. Nuclei were then diluted by addition of 2 volumes of lysis buffer (5 % (v/v) glycerol, 40 mM Tris–HCl pH 7.5, 300 mM KCl, 0.4 mM MgCl_2_, 2 mM DTT, 4 mM Na_3_VO_4_, 10 μg/mL aprotinin, 5 μg/mL leupeptin) and sonicated 4× 30 s; each sonication cycle was followed by 30 s incubation on ice. Lysate was clarified by centrifugation at 14,000× *g* for 10 min, and the resulting supernatant was further centrifuged at 100,000× *g* for 30 min. High molecular weight protein complexes were concentrated using a spin filter concentrator (100,000 MWCO) to reach a protein concentration of approximately 20 mg/mL. A total of 80 μL of this solution was separated using by size-exclusion chromatography (SEC) using a 600 × 7.8 mm BioSep4000 column (Phenomenex, Inc.) operated at 250 μL/min in SEC buffer (5 % (v/v) glycerol, 30 mM Tris–HCl pH 8, 200 mM KCl, 0.3 mM MgCl_2_, 1.7 mM DTT) on a ÄKTA Micro FPLC system (GE). Forty-three fractions (250 μL each) were collect across the column separation range, estimated to be in the range of 2–200 kDa. Urea was added to each fraction to a final concentration of 4 M, and protein were digested by addition of LysC (Wako) (1:100, 4 h at 37 °C) and trypsin (Promega) (1:50, 16 h at 37 °C), following dilution of urea to 2 M. Digestion was stopped by adding TFA to a final concentration of 0.5 % (v/v). Digested peptides were desalted using OASIS C18 96-well plates (Waters) according to manufacturer’s instructions.

Targeted proteomics assays for NuRD members (MBD2, MBD3, MTA1/2/3, and CHD3/4) were developed as described in [57] (Additional file 8). Isotopically labeled peptides corresponding to the selected endogenous peptides (Spike Tides L, JPT) were spiked into each SEC fraction and used as internal standard for quantification. For each fraction, the light-to-heavy ratio of each peptide was normalized to the median ratio of all the NuRD members’ peptides. Normalized ratios were then averaged for each complex member to derive normalized protein intensities that were used for comparison across cell lines (Fig. 5b).

### Induction of an artificial paralog switch in the NuRD complex by silencing MBD3

#### Generation of MBD3 knockdown cell line

Modified human embryonic kidney cells 293 (HEK Flp-In™ T-REx™ 293 cell line, Life Technologies) were grown in Delbecco’s modified Eagle medium (DMEM) containing 5 g/L glucose supplemented with 10 % heat inactivated fetal bovine serum (FBS), blasticidin (15 μg/mL), and zeocin (100 μg/mL). Cell were grown in 37 °C in 5 % CO2. HEK Flp-In™ T-REx™ 293 cells encoding micro-RNA against MBD3 gene were genetically engineered using miRNA BLOCK-iT system from Life Technologies (target: AGATGCTGATGAGCAAGATGA). For stable transfection 200,000 cells were seeded in DMEM with no antibiotics. After 24 h, 100 μL of DMEM (without antibiotics and FBS) with 3 μL X-tremeGENE9 DNA Transfection Reagent (Roche), 100 ng of miRNA containing vector and pOG44 plasmid (Life Technologies) were mixed, incubated 15 min at room temperature and added to cells. Transfected cells were selected by addition of blasticidin (15 μg/mL) and hygromycinB (100 μg/mL). Expression of miRNA was induced for 96 h with 1 mg/mL tetracycline.

#### Quantification of transcript levels by qPCR analysis

Total RNA was isolate with RNAEasy Mini Kit (Qiagen). A total of 500 ng of RNA was reversely transcribed using QuantiTect Reverse Transcription Kit (Qiagen) following the manufacturer protocol. cDNA was diluted 10-fold in water and used as a template for qPCR with Sybr Green PCR Mater Mix. qPCR reaction was performed according to the following protocol: 1× 95 °C – 10 min (DNA denaturation and polymerase activation); 40× 95 °C −15 s (melting), 60 °C – 1 min (annealing/extension). Gene expression was normalized to a glyceraldehyde 3-phosphate dehydrogenase (GAPDH) gene. Selected primers: MBD2 For: AGCCTCAGTTGGCAAGGTAC Rev: GAGGATCGTTTCGCAGTCTC; MBD3 For: CAGCCGGTGACCAAGATTAC Rev: CATGGTCTTGACCAGCTCCT; GAPDH For:GGTCTCCTCTGACTTCAACA Rev: AGCCAAATTCGTTGTCATAC.

#### Quantification of protein abundance changes by targeted proteomics

Changes in NuRD member protein abundances were assessed by targeted proteomics. Nuclei were isolated and processed as described in [57]. Isotopically labeled peptides were spiked-in and used as internal standard for relative quantification between cell lines transfected with miRNA against MBD3 and a scrambled miRNA control (Life Technologies), as described in [10]. For this experiment, additional assays for lamin A/C, lamin B1, and lamin B2 were included in the panel and used for normalization (Additional file 8).

### Classification of normal and colorectal cancer tissues using variable complex members

We obtained large-scale proteomic dataset from tissue samples of normal mucosa, primary colorectal carcinoma, and nodal metastases from Wiśniewski et al. [30]. From the provided MaxQuant output table, we extracted protein intensities used for label-free quantification (LFQ intensities) and retained proteins that were identified by at least two unique peptides. The original dataset contained eight, eight, and seven samples for normal, carcinoma, and metastasis tissue, respectively. We filtered out proteins that were quantified in less than four samples per group. The intensities from the remaining 6148 protein groups were log_2_ transformed and normalized by quantile normalization. We used the nearest-centroid approach to classify cancer versus normal tissues using their proteomic profiles [58]. For the classification purpose, protein features were pre-selected from the list of 53 “variable complex members” that were found to be variable in both reprogramming and 11 cell lines dataset (Additional file 6). Using the leave-one-out method, we evaluated the performance of variable complex members in comparison to random proteins. Variable complex members and random proteins were sampled to generate feature sets while the number of features was in the range of 4–28 in increments of 4. For each size, average accuracy was calculated from 100 sampled features. On average, 20 features from variable complex members were sufficient to classify all the cancer versus normal samples correctly. To highlight the discriminative power of variable complex members in comparison to random (n = 20 features), selected examples were visualized as dendrograms using average linkage hierarchical clustering with Euclidean distance as the similarity measure (Fig. 6b).

### Availability of data and materials

The source codes used are available at http://www.bork.embl.de/Docu/variable_complexes/ under the GNU General Public License v3.0.

The list of the protein complex interfaces with calculated buried surface area accessibilities and HADDOCK/CNS energies and full simulations are available at http://www.bork.embl.de/Docu/variable_complexes/.

The targeted proteomics data for the analysis of NuRD composition in HeLa and HEK293 nuclear extracts and upon MBD3 knockdown are available at http://www.peptideatlas.org/PASS/PASS00792 and http://www.peptideatlas.org/PASS/PASS00793, respectively.

## Additional files

Additional file 1: Document containing supplementary **Figures S1–S5** and their legends. (PDF 1826 kb) Additional file 2: Table listing the protein complex resource. (XLSX 42 kb) Additional file 3: Table containing the microarray data from 10 different cell lines used for the co-expression analysis. (XLSX 51 kb) Additional file 4: Table listing the statistics of quantified protein complexes. (XLSX 48 kb) Additional file 5: Table reporting the functional enrichment analysis of variable and stable protein complexes. (XLSX 45 kb) Additional file 6: Table reporting the summary of quantified complex members and their regulation. (XLSX 179 kb) Additional file 7: Table listing the co-regulated pairs of paralogous proteins. (XLSX 48 kb) Additional file 8: Table listing the targeted proteomics assays used for quantification of NuRD complex members. (XLSX 62 kb)
