# Supplementary material for: Spatiotemporal variation of mammalian protein complex stoichiometries
Source: Genome Biol. 2016 Mar 14;17:47. doi: 10.1186/s13059-016-0912-5 (PMC4791834; doi:10.1186/s13059-016-0912-5)
Supplement: Additional file 1: — Document containing supplementary Figures S1–S5 and their legends. (PDF 1826 kb) [file 13059_2016_912_MOESM1_ESM.pdf]

Supplementary figures to:

Spatiotemporal variation of mammalian protein complex stoichiometries

Alessandro Ori, Murat Iskar, Katarzyna Buczak, Panagiotis Kastritis,

Luca Parca, Amparo Andrés-Pons, Stephan Singer,

Peer Bork, and Martin Beck

Content:

Figures S1:S5

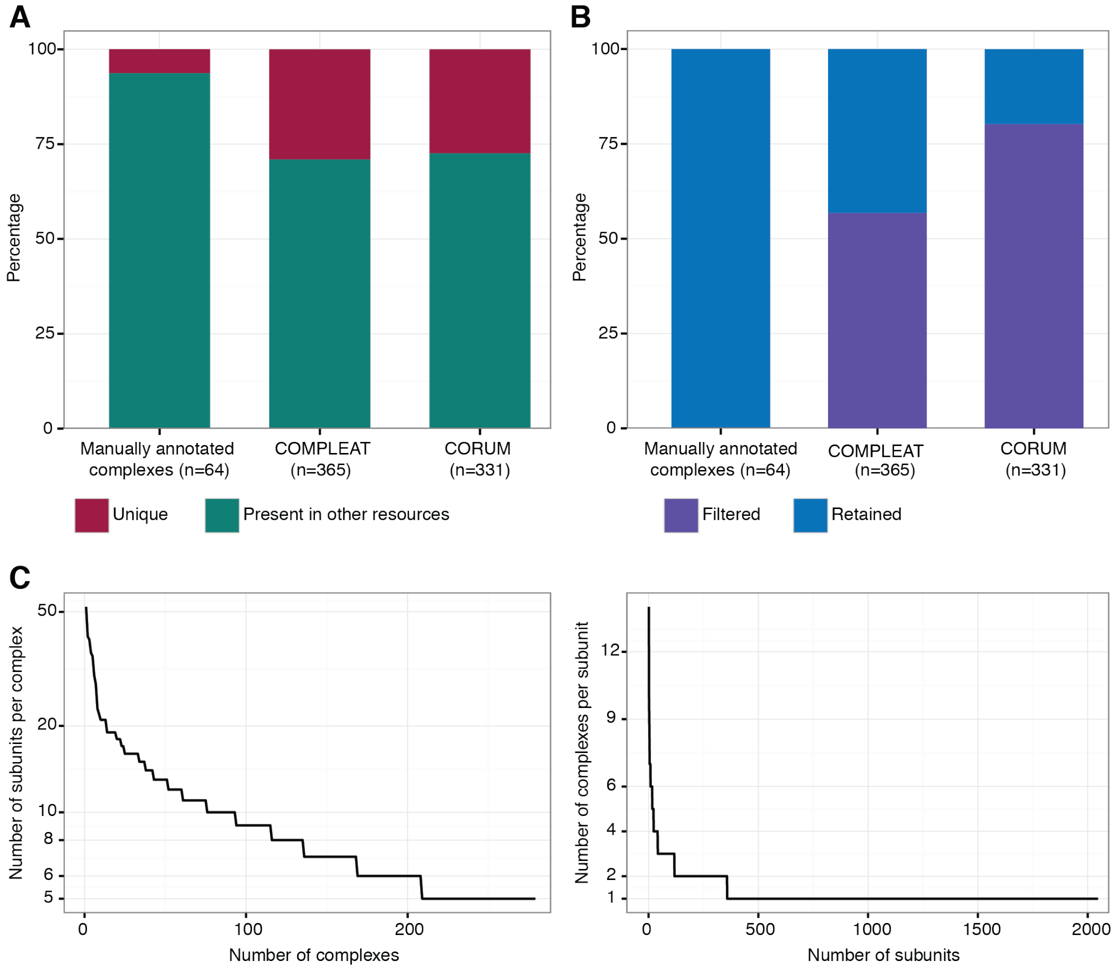

Figure S1. Statistics of protein complex resource. (A) Complex definitions retrieved from independent resources were compared in order to identify redundant complex definitions. A complex was considered to be redundant if another resource had at least one definition sharing half or more complex members. (B) After prioritizing complexes based on their source and size (Methods), we filtered overlapping definitions. The bar chart indicates the percentages of unique complexes retained from each resource following the filtering procedure. (C) The number of protein members per complex (y axis) was plotted against the total number of complexes having that many members (x axis, ordered decreasingly, left panel), and vice versa, the number of complexes per protein was plotted against the total number of distinct proteins (right panel). The left panel shows that there are 93 complexes (33%) with 10 or more members and 208 complexes

(75%) with 6 or more members. The right panel shows that 1,690 out of 2,047 (83%) members were uniquely assigned to a protein complex while 117 members (6%) were shared between 3 or more complexes.

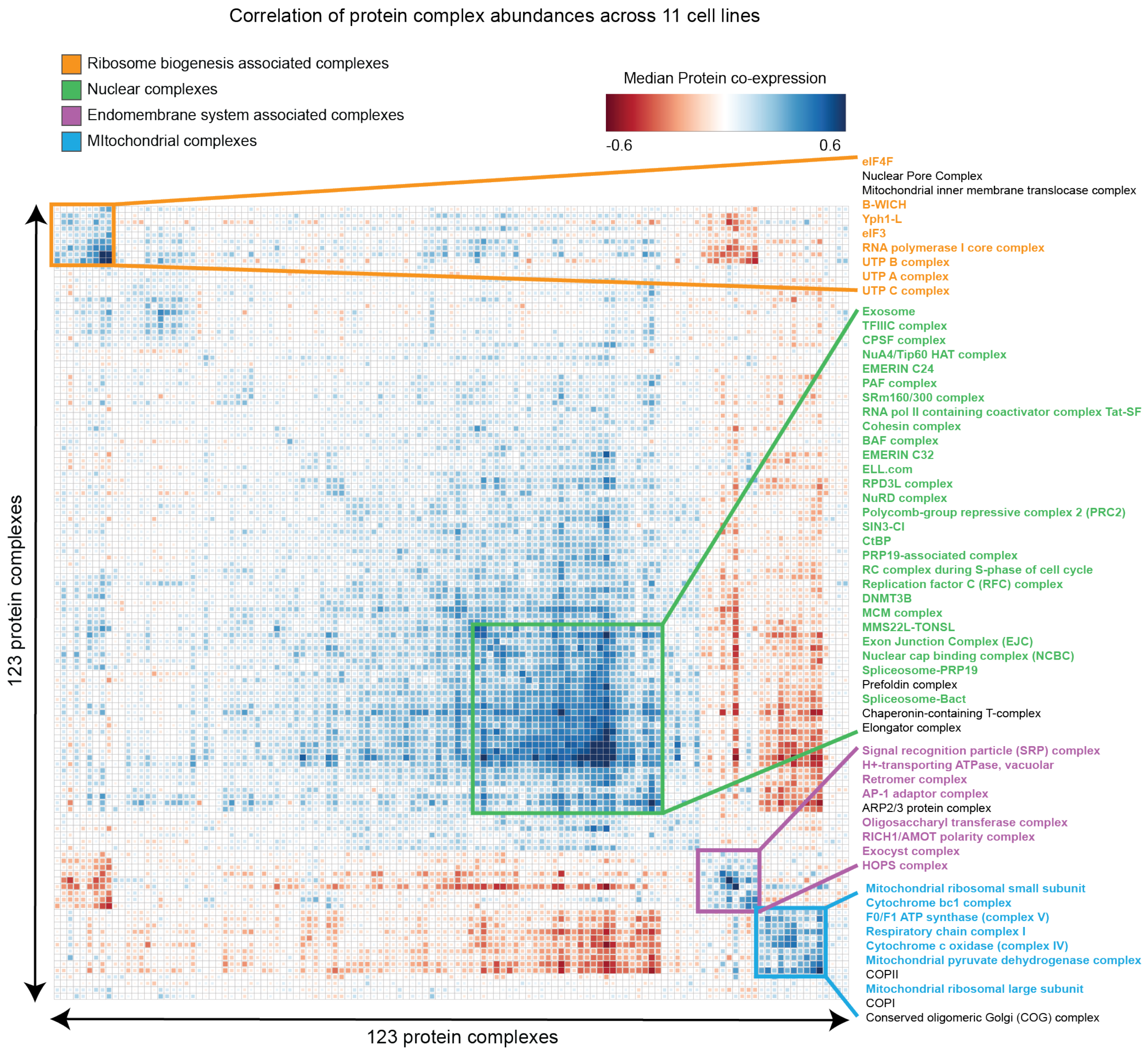

Figure S2. Co-expression analysis clusters together protein complexes from the same cell compartment. We analyzed the correlation between the abundances of 123 large protein complexes (>= 5 subunits) across proteome profiles of 11 cell lines. For each pair of protein complexes, we calculated a co-expression value by taking the median of all pairwise comparisons between members of the protein complexes. Co-expression matrix of the protein complexes was represented as a heatmap using the R package *corrplot*. Protein complexes were ordered by unsupervised hierarchical clustering with average

linkage. Protein complexes that form highly correlated clusters tend to co-localize to the same cell compartment. Ribosome biogenesis associated complexes are shown in orange, nuclear complexes in green, endomembrane system associated complexes in purple and mitochondrial complexes in blue.

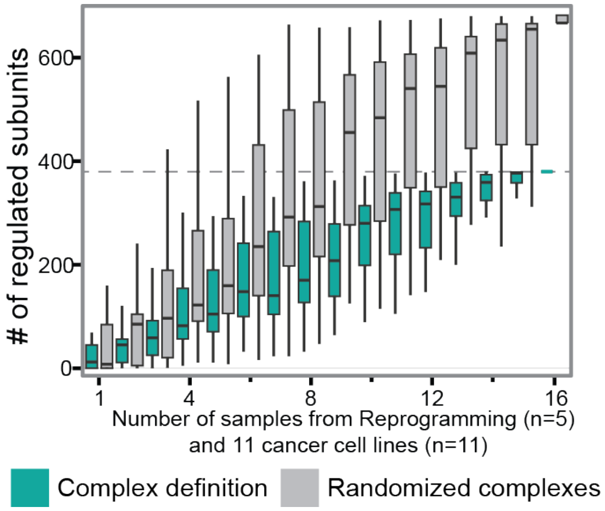

Figure S3. Saturation curve of variable complex members. For reprogramming and 11 cancer cell line datasets, ten random sets of protein complexes were generated from the same pool of protein members. In this process, the total number and the size of the complexes were constrained to be the same as the original complex definitions. Both for the original (green) and ten randomized set (grey) of protein complexes, the number of distinct variable members was calculated for the random subset (from 1 to 16) of 16 condition(s) (n=10). The boxplots show a tendency towards saturation of the number of variable complex members for protein complexes in comparison to randomized definitions.

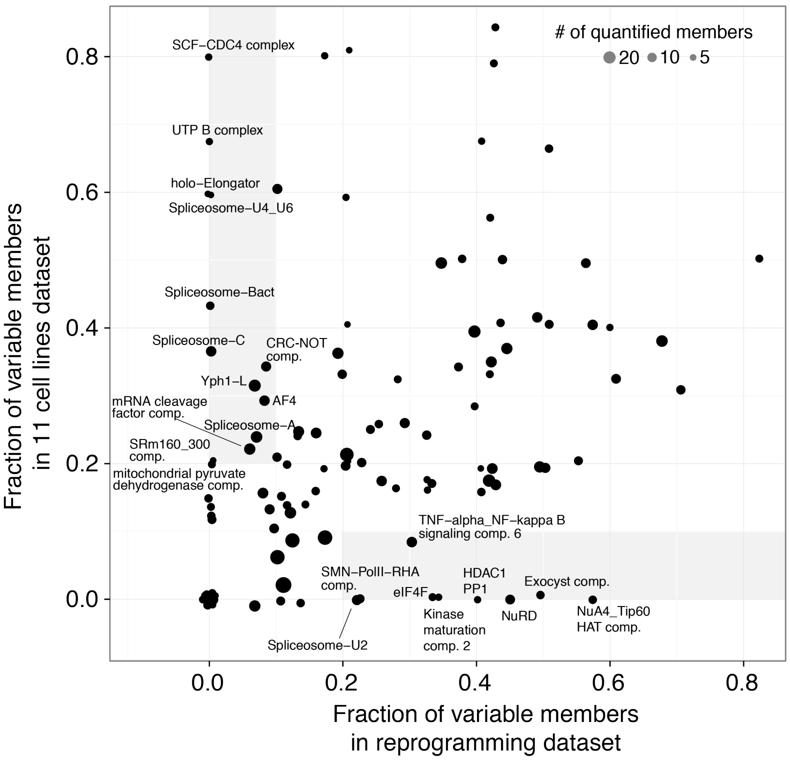

Figure S4. The fraction of variable complex members correlates between the reprogramming and 11 cell lines dataset with some exceptions. For each of the 116 complexes quantified in both datasets, the fraction of variable complexes in the reprogramming and 11 cell lines dataset is compared. The fraction of variable members generally correlates between the two datasets, however some complexes were identified as variable only in one of them (>=0.2 in one dataset and <0.1 in the other dataset, labeled as grey). These examples are labeled with the complex name. The dot size indicate the total number of complex members quantified in at least one dataset (comp. : complex).

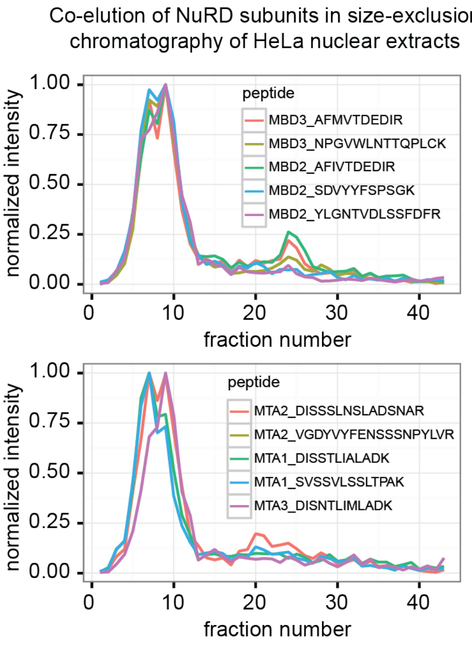

Figure S5. Isolation of endogenous NuRD complex from nuclear extracts. We separated nuclear extracts from the two cell lines by size-exclusion chromatography and quantified the elution of NuRD components using targeted proteomics (Methods). The graphs show that peptides deriving from the NuRD components are detected in the same fractions following size-exclusion chromatography. This indicates co-elution of the NuRD components. The depicted peptides have been used to quantify the relative abundance of MBD2 and MBD3 between HeLa and HEK293 cells (Figure 5). For display purpose, the intensities of different peptides have been normalized to their maximum value in this graph.
